# Coronary artery calcification and aortic valve calcification in patients with kidney failure: a sex-disaggregated study

**DOI:** 10.1186/s13293-023-00530-x

**Published:** 2023-07-13

**Authors:** Liam J. Ward, Agne Laucyte-Cibulskiene, Leah Hernandez, Jonaz Ripsweden, GOING-FWD Collaborators, Louise Pilote, Colleen M. Norris, Valeria Raparelli, Alexandra Kautzky-Willer, Maria Trinidad Herrero, Peter Stenvinkel, Karolina Kublickiene

**Affiliations:** 1 Division of Renal Medicine, Department of Clinical Science, Intervention and Technology, Karolinska Institutet, 141 86 Stockholm, Sweden; 2 Department of Forensic Genetics and Forensic Toxicology, National Board of Forensic Medicine, Linköping, Sweden; 3 Department of Nephrology, Lund University, Skåne University Hospital, Malmö, Sweden; 4 Unit of Radiology, Department of Clinical Science, Intervention and Technology, Karolinska Institutet, Stockholm, Sweden; 5 Department of Radiology, Karolinska University Hospital, Huddinge, Stockholm, Sweden

**Keywords:** Calcification, Calcific aortic valve disease, Cardiovascular disease, Chronic kidney disease, Inflammation, Oxidative stress, Vascular remodelling

## Abstract

**Background:**

Chronic kidney disease (CKD) is linked to an increased cardiovascular disease (CVD) burden. Albeit underappreciated, sex differences are evident in CKD with females being more prone to CKD development, but males progressing more rapidly to kidney failure (KF). Cardiovascular remodelling is a hallmark of CKD with increased arterial and valvular calcification contributing to CKD. However, little is known regarding sex differences in calcific cardiovascular remodelling in KF patients. Thus, we hypothesise that sex differences are present in coronary artery calcification (CAC) and aortic valve calcification (AVC) in patients with KF.

**Methods:**

KF patients, males (n = 214) and females (n = 107), that had undergone computer tomography (CT) assessment for CAC and AVC were selected from three CKD cohorts. All patients underwent non-contrast multi-detector cardiac CT scanning, with CAC and AVC scoring based on the Agatston method. Baseline biochemical measurements were retrieved from cohort databases, including plasma analyses for inflammation markers (IL-6, TNF, hsCRP) and oxidative stress by skin autofluorescence measuring advanced glycation end-products (AGE), amongst other variables.

**Results:**

Sex-disaggregated analyses revealed that CAC score was associated with age in both males and females (both p < 0.001). Age-adjusted analyses revealed that in males CAC was associated with diabetes mellitus (DM) (p = 0.018) and CVD (p = 0.011). Additionally, for females CAC associated with IL-6 (p = 0.005) and TNF (p = 0.004). In both females and males CAC associated with AGE (p = 0.042 and p = 0.05, respectively). CAC was associated with mortality for females (p = 0.015) independent of age. AVC in females was not reviewed due to low AVC-positive samples (n = 14). In males, in multivariable regression AVC was associated with age (p < 0.001) and inflammation, as measured by IL-6 (p = 0.010).

**Conclusions:**

In female KF patients inflammatory burden and oxidative stress were associated with CAC. Whereas in male KF patients oxidative stress and inflammation were associated with CAC and AVC, respectively. Our findings suggest a sex-specific biomarker signature for cardiovascular calcification that may affect the development of cardiovascular complications in males and females with KF.

## Plain language summary

Chronic kidney disease (CKD) is a condition that affects the kidneys and increases the risk of heart problems. Males and females may experience CKD differently, and our study aimed to understand the differences in the development of calcification in the blood vessels of the heart (coronary artery calcification, or CAC) and the heart valves (aortic valve calcification, or AVC) between males and females with CKD.

We analysed 214 males and 107 females with CKD who had undergone a heart scan (computer tomography, or CT) to measure CAC and AVC. We collected information on age, diabetes, cardiovascular disease, and markers of inflammation and oxidative stress.

Our results showed that in both males and females CAC was associated with age. In males, CAC was associated with diabetes and cardiovascular disease, while in females, it was linked to markers of inflammation. In females, CAC was also associated with mortality regardless of age. Unfortunately, we had insufficient samples of females with AVC for analysis. However, in males AVC was associated with age and inflammation.

Overall, our study indicates sex-specific differences in the development of calcification in the blood vessels and heart valves of CKD patients. In females, inflammation and oxidative stress are associated with CAC, while in males, oxidative stress and inflammation are associated with CAC and AVC, respectively. These findings underscore the importance of considering these differences when assessing cardiovascular complications in CKD patients. It may help in developing personalised treatment approaches for both males and females with CKD.

## Highlights

- The gold standard technique of computer-tomography was used to measure coronary artery calcification (CAC) and aortic valve calcification (AVC) scores in males and females with kidney failure.
- In male kidney failure patients age and inflammatory burden was significantly associated with AVC. In addition, cardiovascular disease, diabetes, and oxidative stress were significantly associated with CAC score.
- In female kidney failure patients age, oxidative stress, and inflammation were significantly associated with CAC score.
- Cardiovascular calcification presents with a sex-specific biomarker signature that may affect cardiovascular complications in males and females with kidney failure.

## Background

Chronic kidney disease (CKD) is a debilitating disease that has become a worldwide health care burden affecting foremost individuals with burden of lifestyle diseases, such as diabetes and hypertension [1]. CKD is linked to an increased risk for cardiovascular disease (CVD) and mortality, with the elderly and females more prone to CKD development [2]. However, CKD in males tends to progress more rapidly toward kidney failure (KF), which highlights the important influence of sex on CKD phenotype.

Cardiovascular remodelling via vascular and/or valvular calcification is a key hallmark of CKD-mineral and bone disorders (CKD-MBD) [3]. CKD-MBD contributes to accelerated uraemia-induced vascular ageing [4] by potentiating cellular senescence [5], modifying the function and structure of vascular endothelial and vascular smooth muscle cells (VSMC), which includes effects on innate immunity [5].

Vascular calcification, per se, is an active process that manifests as early as kidney function starts to deteriorate [6] and is influenced by both traditional and non-traditional cardiovascular risk factors [7]. Potential CKD-related mechanisms behind the simultaneous tunica intima (atherosclerosis) and tunica media (arteriosclerosis) calcification, alongside calcific aortic valve disease (CAVD), have been described in previous studies [8–10]. However, the identification of biological sex-related factors in this population is still lacking. This is of importance, as sex as a biological variable could modulate several triggering processes associated with vascular calcification, including inflammation, imbalanced calcium-phosphate metabolism, VSMC damage and its associated phenotypic switch towards the activation of bone formation pathways.

Thus, the rationale behind our study was to assess sex-specific differences in cardiovascular calcification measured at the different sites. We aimed to analyse associations between CAC and AVC, evaluated via computed tomography (CT) scans, with comorbidities and cardiovascular, glycaemic, and oxidative stress biomarkers in a population of males and females with kidney failure. The hypothesis is that males with kidney failure will have greater levels of CAC and AVC compared to that of females with kidney failure.

## Methods

### Study population

For the current investigation post hoc analyses of data collected from 321 KF patients that had undergone CT scans to assess CAC and AVC, were included. Patient data were included from three prospective CKD cohort databases from the Division of Renal Medicine, Karolinska University Hospital, Sweden. Patients with KF were included from either an incident CKD cohort [11], a prevalent CKD cohort with peritoneal dialysis (PD) treatment [12], or a living-donor kidney transplantation (LD-Tx) cohort [9]. The incident CKD cohort is a prospective cohort, with ongoing recruitment since 1994, of patients with kidney failure (CKD stage 5, GFR < 15 ml/min) with baseline sampling occurring close to the initiation of renal replacement therapy (RRT), with the vast majority having started haemodialysis or PD [11]. For the current study, data were collated for patients included between February 2006 and December 2014 from the incident KF cohort, where CT scans were performed. The prevalent CKD cohort was a prospective cohort, with recruitment between March 2008 and April 2011, including CKD patients undergoing PD. The LD-Tx cohort is a prospective cohort, with ongoing recruitment since March 2009, of kidney failure patients undergoing kidney transplantation. The available study population consisted of 214 males and 107 females, and all data previously included in the cohort databases, either derived from patient records or prior experimental results, were made available for analysis in relation to CAC and AVC.

The Regional Ethics Committee (EPN), Stockholm, Sweden, approved the study protocols that were performed in accordance with the Declaration of Helsinki. Written informed consent was obtained from all patients involved in the study.

Clinical data were recorded at baseline, during the first visit, and included information on demographics, medications, comorbidities (CVD and diabetes mellitus), smoking status, and malnutrition that was assessed by subjective global assessment (SGA). eGFR collected for this study was calculated using the creatinine-based CKD-EPI 2009 Equation [13], validated in the European population. Mortality was documented over a five year follow up period among KF patients. Death cases were retrieved either from patient’s medical journal or the National cause of death register.

### Biochemical measurements

Baseline data for biochemicals were retrieved from the cohort databases for all patients included in the current investigation, where available. Biochemical measurements were recorded using overnight fasting blood samples that were collected in the morning, with serum isolated for necessary analyses, and samples were either immediately analysed or stored at -70 °C for future analyses. Analyses for blood lipids, haemoglobins, albumin, creatinine, and inflammation markers (IL-6, hsCRP, TNF) were performed via routine clinical laboratory techniques. Cardiovascular disease (CVD) risk markers (GDF-15, MMP-9, YKL-40) were analysed using commercial enzyme-linked immunosorbent (ELISA) kits, previously described [14]. Skin autofluorescence was measured as a proxy of advanced glycation end-products (AGE), using an Autofluorescence AGE reader (DiagnOptics Technologies BV, the Netherlands) as previously described [15].

### Coronary artery calcification and aortic valve calcification

All patients underwent non-contrast multi-detector cardiac CT (LightSpeed VCT or Revolution CT; GE Healthcare, WI, USA) scanning with standard ECG-gated protocol, to evaluate AVC and CAC scores. We used semi-automatic software (syngo.via CT Ca Scoring, Siemens Healthcare, Germany). CAC score was assessed as a lesion with an area > 1 mm^2^and peak intensity > 130 Hounsfield units (HU) based on the Agatston method and expressed in Agatston units (AU) [16]. AVC scores were computed using the Agatston CAC-scoring method from non-contrast cardiac CT scans. AVC score was determined as the sum of total calcifications in the aortic valve area including calcifications within the valve leaflets as well as in the aortic wall immediately connected to the leaflets.

### Statistical analyses

Continuous data are expressed as median ± interquartile range, and categorical data are presented as the frequency with percentage. Individual counts for analytes are provided to present the number of patients with available data. Baseline comparisons between females and males, for continuous data Wilcoxon sum rank test was performed, and for categorical data either Fisher’s exact test or Pearson’s Chi-squared test were performed.

This investigation was designed as a sex-disaggregated study, therefore further analysis was performed after the sub-setting the data set by sex, one for males and the other for females. First, all analytes were separately put in the sex-disaggregated univariate Gamma regression by using CAC or AVC scores as dependent variables. Gamma regression, using the identity link, analyses were performed to assess associations between independent variables and CAC scores. The Variance Inflation Factors (VIF) tool was used to address multicollinearity within multivariable regression analyses assessing CAC scores. Herein, multivariable models that resulted in variables with a VIF greater then 2, indicating a high collinear relationship to other variables, were not interpreted. Multivariable models were reduced in terms of number of variables included so that collinearity was not present in accordance with VIF.

For analysing AVC score we had to perform Gamma regression analyses using the log link to solve convergence issues. The values were displayed as relative rates using inverse transformation of each value. Then, multivariable regression was performed by adjusting only significantly associated variables (*p-*value < 0.05).

After performing sex-disaggregated logistic regression analyses, all significant variables were put into multivariate regression to assess associations between mortality and CAC scores. The best model was selected using the Bayesian Information Criterion and included age, systolic blood pressure, plasma albumin, CAC score and GDF-15 (only in males). All statistical analyses were carried out using R (v4.1.3) in the RStudio environment (RStudio, MA, USA).

## Results

### Study population and baseline characteristics

Patients with KF enrolled in the study included 214 males and 107 females with baseline characteristics presented in Table 1. As expected, males presented with a greater frequency of cardiovascular disease comorbidity, and this is presumed why males were more frequently treated with beta-blockers when compared to females (Table 1). Females had significantly elevated cholesterol, high-density lipoprotein (HDL), lipoprotein(a), and apolipoprotein-A1 when compared to males (Table 1). Additionally, females presented with a higher frequency of protein energy wasting, assessed via SGA, and lower albumin levels than males (Table 1). As expected, males had higher creatinine levels than females, likely a generalised reflection of greater muscle mass (Table 1).

**Table 1:** Clinical, laboratory, and imaging characteristics of the study population of patients with kidney failure stratified by sex

	Males, n = 214^1^	Females, n = 107^1^	p-value^2^
Clinical data			
Age, years	52 (41–65)	55 (45–64)	0.39
Body mass index, kg/m^2^	25.1 (23.0–27.8), n = 211	24.4 (21.4–28.0), n = 105	0.11
Systolic blood pressure, mmHg	143 (128- 160), n = 203	138 (125–153), n = 101	0.22
Diastolic blood pressure, mmHg	87 (79–95), n = 203	86 (78–94), n = 105	0.56
Cardiovascular disease	36 (27%), n = 135	4 (5.6%), n = 71	** < 0.001**
Diabetes mellitus	32 (19%), n = 172	14 (16%), n = 90	0.61
Renal replacement therapy (RRT)	92 (45%), n = 201	40 (38%), n = 105	0.20
Haemodialysis	31 (34% of RRT)	10 (25% of RRT)	
Peritoneal dialysis	44 (48% of RRT)	28 (70% of RRT)	
Other (incl. Tx)	17 (18% of RRT)	2 (5% of RRT)	
Vintage, years	1.1 (0.5–3.0), n = 41	1.0 (0.8–2.0), n = 19	0.60
eGFR, ml/min/1.73^2^	6.21 (5.19–8.57), n = 110	6.51 (4.64–8.40), n = 57	0.92
Smoking	10 (8.7%), n = 115	4 (6.2%), n = 65	0.77
Malnutrition, SGA > 1	63 (32%), n = 199	45 (44%), n = 103	**0.039**
Medications at cohort entry			
ACE-inhibitors/ARBs	124 (67%), n = 186	58 (65%), n = 89	0.89
Beta-blockers	129 (69%), n = 186	48 (54%), n = 89	**0.015**
Ca^2+^ channel blockers	123 (66%), n = 186	49 (55%), n = 89	0.084
Statins	74 (40%), n = 186	31 (35%), n = 89	0.51
Biochemicals			
Cholesterol, mmol/L	4.3 (3.6–4.9), n = 213	4.8 (4.0–5.6), n = 104	** < 0.001**
HDL, mmol/L	1.2 (1.0–1.5), n = 213	1.5 (1.2–1.7), n = 104	** < 0.001**
Triglycerides, mmol/L	1.5 (1.1–2.0), n = 213	1.6 (1.2–2.2), n = 104	0.39
Lipoprotein(a), mmol/L	79.5 (30.5–204.3), n = 156	123.0 (46.3–421.8), n = 82	**0.038**
Apolipoprotein-A1, g/L	1.32 (1.14–1.52), n = 211	1.52 (1.36–1.71), n = 105	** < 0.001**
Apolipoprotein-B, g/L	0.83 (0.66–1.00), n = 211	0.89 (0.72–1.07), n = 105	0.054
Creatinine, µmol/L	757 (622–911), n = 213	629 (509–740), n = 105	** < 0.001**
Albumin, g/L	35.0 (31.0–38.0), n = 212	33.0 (30.8–36.0), n = 104	**0.018**
Haemoglobin, g/L	113.0 (103.5–120.0), n = 147	112.0 (101.8–120.0), n = 84	0.64
HbA1c, mmol/mol	33.0 (28.0–38.0), n = 205	34.0 (28.0–38.0), n = 104	0.85
Calcium, mmol/L	2.3 (2.2–2.4), n = 212	2.3 (2.2–2.4), n = 105	0.22
Phosphate, mmol/L	1.8 (1.5–2.2), n = 212	1.7 (1.5–1.9), n = 105	0.19
25-OH vitamin D, nmol/L	33.0 (14.0–53.5), n = 187	30.0 (12.0–54.3), n = 84	0.59
1,25 vitamin D, pmol/L	17.0 (12–23.5), n = 84	17.0 (14.3–22.8), n = 46	0.71
hs-CRP, mg/L	1.2 (0.6–3.6), n = 181	1.2 (0.5–3.5), n = 88	0.65
TNF, pg/mL	12.1 (9.3–16.5), n = 88	12.4 (10.1–16.2), n = 54	0.63
IL-6, pg/mL	2.3 (1.0–6.4), n = 78	3.4 (1.4–7.4), n = 50	0.42
AGE, skin AF	3.1 (2.5–3.5), n = 98	3.4 (2.8–4.0), n = 48	**0.021**
Homocysteine, µmol/L	36.0 (28.3–50.8), n = 110	29.0 (22.8–40.3), n = 44	**0.002**
GDF-15, ng/mL	4.3 (2.9–5.2), n = 118	4.4 (3.3–5.2), n = 62	0.86
MMP-9, ng/mL	381.1 (244.2–574.8), n = 116	363.3 (229.5–641.1), n = 62	0.88
YKL-40, ng/mL	108.3 (77.5–161.0), n = 117	119.8 (89.6–184.6), n = 60	0.27
Imaging markers			
CAC score, AU (ln + 1)	4.16 (0.00–6.88), n = 212	3.86 (0.00–5.94)	0.11
AVC score, AU (ln + 1)	0.00 (0.00–0.69), n = 209	0.00 (0.00–0.00)	**0.005**
Follow-up, 5 years			
All-cause mortality	20 (11%), n = 177	11 (12%), n = 92	0.84

Bold p-values signify significance

*ACE* angiotensin-converting enzyme, *AF* autofluorescence, *AGE* advanced glycation end-products, *ARB* angiotensin receptor-blockers, *AVC* aortic valve calcification, *CAC* coronary artery calcification, *hsCRP* high sensitivity C-reactive protein, *eGFR* estimated glomerular filtration, *GDF-15* growth differentiation factor 15, *HbA1c* glycated haemoglobin, *HDL* high-density lipoprotein, *IL-6* interleukin-6, *MMP-9* matrix metalloproteinase 9, *SGA* subjective global assessment, *TNF* tumour necrosis factor, *YKL-40* chitinase-3-like protein 1

^1^Median (IQR); n (%); ^2^Wilcoxon rank sum test; Fisher's exact test; Pearson's Chi-squared test

### Sex-specific CAC score associations with age, comorbidities, inflammation, and oxidative stress

In linear regression analyses, age was significantly associated with CAC scores in both males and females (Table 2). Age-adjusted linear regressions were performed for all other available variables, with age remaining significantly associated with CAC score in all models for both males and females. More extensive multivariable linear regressions were not possible due to increasing collinearity, as tested via the VIF tool, between additional variables that were included in models. For example, when for analysing predictive factors for CAC scores in males, we could not interpret a model including age (VIF = 54), CVD (VIF = 1), DM (VIF = 1), use of statins (VIF = 44), cholesterol (VIF = 9), AGE (VIF = 2) and homocysteine (VIF = 9) due to the high VIFs. In females, all VIFs were above 2 in multivariate Gamma regression.

**Table 2:** Sex-divided, age-adjusted, gamma regression models showing significant associations of coronary artery calcification (CAC) with comorbidities, inflammation, and oxidative stress markers in patients with kidney failure (KF)

CAC score	Males (n = 214)			Females (n = 107)		
	Estimate	95% CI	p-value	Estimate	95% CI	p-value
Age^†^	18.62	14.51–22.73	** < 0.001**	11.27	6.72–15.82	** < 0.001**
Body mass index, kg/m^2^	-0.02	− 0.41–0.37	0.918	− 3.58	− 5.70 to − 1.46	**0.001**
SBP, mmHg	0.001	− 0.05–0.06	0.959	− 0.35	− 0.54 to − 0.15	**0.001**
CVD, yes	1143.3	204.6–2081.9	**0.018**	805.21	− 1807.5–3418.0	0.548
DM, yes	1188.7	286.6–2090.8	**0.011**	874.84	− 671.2–2420.8	0.270
Renal replacement therapy	0.05	− 2.54–2.64	0.968	12.52	5.46–19.59	**0.001**
eGFR, ml/min/1.73^2^	− 0.002	− 0.51–0.51	0.993	− 2.005	− 3.37 to − 0.64	**0.006**
Malnutrition, SGA > 1	0.01	− 2.40 to − 2.42	0.995	− 11.48	− 17.86 to − 5.10	**0.001**
ACE-inhibitors/ARBs	− 0.02	− 2.71–2.67	0.988	− 8.49	− 14.29 to − 2.68	**0.005**
Beta-blockers	− 0.00	− 2.69–2.69	0.999	8.49	2.72–14.26	**0.005**
Statins	− 135.18	− 170.55 to − 99.81	** < 0.001**	155.33	− 170.18–480.83	0.352
Cholesterol, mmol/L	− 13.14	− 16.27 to − 10.00	** < 0.001**	6.18	2.08–10.28	**0.004**
HDL, mmol/L	0.02	− 2.36–2.40	0.988	55.99	22.54–89.43	**0.001**
Triglycerides, mmol/L	0.04	− 1.91–2.00	0.966	− 44.21	− 67.61 to − 20.82	** < 0.001**
Lipoprotein(a), mmol/L	− 0.00	− 0.01–0.01	0.999	0.09	0.02–0.16	**0.012**
Apolipoprotein-A1, g/L	− 0.05	− 4.09–4.00	0.982	156.64	89.13–224.14	** < 0.001**
Apolipoprotein-B, g/L	− 0.73	− 8.11–6.65	0.847	19.11	7.05–31.18	**0.003**
Creatinine, µmol/L	− 0.00	− 0.01–0.01	0.999	0.04	0.02–0.06	**0.001**
Albumin, g/L	0.01	− 0.48–0.50	0.962	− 1.33	− 2.22 to − 0.44	**0.004**
Haemoglobin, g/L	0.00	− 0.08–0.08	0.994	− 2.50	− 3.99 to − 1.01	**0.001**
HbA1c, mmol/mol	0.00	− 0.13–0.13	1.000	0.72	0.28–1.16	**0.002**
hs-CRP, mg/L	0.09	− 2.32–2.50	0.942	6.24	1.99–10.49	**0.005**
TNF, pg/mL	0.02	− 0.43–0.47	0.948	1.16	0.41–1.91	**0.004**
IL-6, pg/mL	68.39	0.48–136.29	0.052	3.73	1.13–5.62	**0.005**
AGE, skin AF	16.03	5.21–26.84	**0.005**	221.21	13.78–428.64	**0.042**
Homocysteine, µmol/L	− 0.22	− 0.33 to − 0.12	** < 0.001**	− 0.32	− 0.60 to − 0.03	**0.034**
GDF-15, ng/mL	0.03	− 1.26–1.33	0.960	− 1.61	− 2.84 to − 0.38	**0.013**
MMP-9, ng/mL	− 0.00	− 0.00–0.00	0.996	0.02	0.01–0.04	**0.014**
YKL-40, ng/mL	0.00	− 0.05–0.05	0.979	− 0.28	− 0.51 to − 0.05	**0.021**

Each predictor was modelled separately together with age as a covariate

Bold p-values signify significance.

*ACE* angiotensin-converting enzyme, *AF* autofluorescence, *AGE* advanced glycation end-products, *ARB* angiotensin receptor-blockers, *AVC* aortic valve calcification, *CAC* coronary artery calcification, *CVD* cardiovascular disease, *DM* diabetes mellitus, *hsCRP* high sensitivity C-reactive protein, *eGFR* estimated glomerular filtration, *GDF-15* growth differentiation factor 15, *HbA1c* glycated haemoglobin, *HDL* high-density lipoprotein, *IL-6* interleukin-6, *MMP-9* matrix metalloproteinase 9, *SBP* systolic blood pressure, *SGA* subjective global assessment, *TNF* tumour necrosis factor, *YKL-40* chitinase-3-like protein 1

^†^Gamma regression (identity link) model with age alone given as a reference

Therefore, any significant biomarker correlation is not an independent association, but coincides with the significant association of age. DM and CVD were associated with CAC scores in males, with HbA1c only being associated with CAC scores in females (Table 2). Other predictive parameters for CAC score in females were body mass index (BMI), systolic blood pressure (SBP), RRT, malnutrition, use of beta-blockers, and ACE inhibitors/ARBs; whereas in males—only with the use of statins. Inflammatory markers hs-CRP, IL-6 and TNF were associated with CAC score in females only, whereas oxidative stress marker AGE was associated with CAC score in both males and females. Lipid profile was significantly correlated to CAC score only in females, alongside additional cardiovascular biomarkers GDF-15, MMP-9, and YKL-40.

### AVC score associations with age and IL-6 in male patients with KF

Gaussian regression analyses for possible associations with AVC score were also performed. Regression models for females were not performed due to the low number of females with an AVC score > 0 (n = 14). In males, the AVC score was significantly associated with age, haemodynamic parameters, lipid status, antihypertensive medication, inflammatory and cardiovascular biomarkers (Table 3). However, after multiple adjustments, the AVC score remained significantly associated with IL-6 and age only.

**Table 3:** Gamma regression models showing significant associations of aortic valve calcification (AVC) score with age and inflammation in male patients with kidney failure (KF)

AVC score	Males (n = 209)					
	Univariate			Multivariate^†^		
	Estimate	95% CI	p-value	Estimate	95% CI	p-value
Age	1.14	1.11–1.17	** < 0.001**	1.29	1.07–1.14	** < 0.001**
Body mass index, kg/m^2^	0.89	0.84–0.94	** < 0.001**	–	–	–
SBP, mmHg	0.96	0.95–0.97	** < 0.001**	–	–	–
DBP, mmHg	0.93	0.90–0.97	** < 0.001**	–	–	–
DM, yes	0.11	0.07–0.16	** < 0.001**	–	–	–
Renal replacement therapy	10.42	6.80–16.00	** < 0.001**	–	–	–
Malnutrition, SGA > 1	0.10	0.06–0.17	** < 0.001**	–	–	–
ACE-inhibitors/ARBs	17.63	9.96–31.20	** < 0.001**	–	–	–
Beta-blockers	22.65	12.52–40.97	** < 0.001**	–	–	–
Ca^2+^ channel blockers	2.80	0.93–8.48	0.070	–	–	–
Statins	0.47	0.20–1.11	0.085	–	–	–
Cholesterol, mmol/L	5.04	3.64–6.98	** < 0.001**	–	–	–
HDL, mmol/L	0.18	0.12–0.29	** < 0.001**	–	–	–
Triglycerides, mmol/L	0.41	0.31–0.53	** < 0.001**	–	–	–
Lipoprotein(a), mmol/L	0.99	0.997 -0.998	** < 0.001**	–	–	–
Apolipoprotein-A1, g/L	0.06	0.03–0.12	** < 0.001**	–	–	–
Creatinine, µmol/L	1.01	1.005 -1.008	** < 0.001**	–	–	–
Haemoglobin, g/L	0.86	0.85–0.88	** < 0.001**	–	–	–
TNF, pg/mL	0.90	0.80–1.02	0.109	–	–	–
IL-6, pg/mL	2.61	2.29–2.98	** < 0.001**	1.16	1.04–1.29	**0.010**
AGE, skin AF	2.56	0.86–7.62	0.093	–	–	–
GDF-15, ng/mL	4.47	3.62–5.52	** < 0.001**	–	–	–
YKL-40, ng/mL	1.02	1.02–1.03	** < 0.001**	–	–	–

Bold p-values signify significance

Gamma regression model (log link), values are presented as relative rates using inverse transformation of each value

*ACE* angiotensin-converting enzyme, *AF* autofluorescence, *AGE* advanced glycation end-products, *ARB* angiotensin receptor-blockers, *AVC* aortic valve calcification, *DBP* diastolic blood pressure, *DM* diabetes mellitus, *GDF-15* growth differentiation factor 15, *HDL* high-density lipoprotein, *IL-6* interleukin-6, *SBP* systolic blood pressure, *SGA* subjective global assessment, *TNF* tumour necrosis factor, *YKL-40* chitinase-3-like protein 1

^†^Multivariate analysis includes all those significant variables presented in the univariate analyses

### CAC score is associated with mortality in females with KF

In multivariate logistics regression analyses, CAC score was shown to be associated with mortality in female patients only, independent of age. Moreover, in multivariable analyses a significant association with albumin was also present for female patients. In males, CAC score was associated with mortality in univariate analyses, however, this association was lost in multivariate analyses. GDF-15 was the only marker to remain significantly associated with mortality in males in the multivariate model. Other significant variables that predicted mortality are listed in Table 4.

**Table 4:** Coronary artery calcification (CAC) is significantly associated with mortality in female kidney failure patients, independent of age

Mortality	Males (n = 177)			Females (n = 92)		
	Univariate			Univariate		
	Odds ratios	95% CI	p-value	Odds ratios	95% CI	p-value
Age	1.10	1.10–1.14	**0.001**	1.10	1.03–1.18	**0.004**
SBP, mmHg	0.96	0.93–0.98	**0.001**	0.97	0.94 -1.00	**0.082**
DM, yes	2.67	0.96–7.33	0.061	10.35	2.56–41.7	**0.001**
Smoking, yes	14.10	2.45–80.9	**0.003**	n/a	n/a	n/a
Albumin, g/L	0.89	0.82–0.97	**0.011**	0.81	0.69–0.94	**0.007**
IL-6, pg/mL	1.15	1.01–1.32	**0.036**	n/a	n/a	n/a
AGE, skin AF	2.67	1.20–5.97	**0.017**	1.53	0.82–2.84	0.184
GDF-15, ng/mL	1.22	1.04–1.45	**0.018**	n/a	n/a	n/a
ln(CAC + 1)	1.64	1.24–2.18	**0.001**	1.66	1.15–2.39	**0.007**

	Multivariate^†^			Multivariate^†^		
	Odds ratios	95% CI	p-value	Odds ratios	95% CI	p-value
Age	1.01	0.88–1.15	0.906	1.05	0.97–1.13	0.276
SBP, mmHg	1.05	0.98–1.11	0.149	0.97	0.94–1.00	0.067
Albumin, g/L	0.93	0.72–1.21	0.601	0.75	0.60–0.95	**0.019**
GDF-15, ng/mL	1.35	1.03–1.77	**0.032**	n/a	n/a	n/a
ln(CAC + 1)	1.21	0.71–2.05	0.486	1.76	1.12–2.78	**0.015**

Bold p-values signify significance. n/a signifies a lack of appropriate datapoints to perform analysis

Logistic regression was performed using logarithmic coronary artery calcification expressed as ln(CAC + 1)

*AF* autofluorescence, *AGE* advanced glycation end-products, *CAC* coronary artery calcification, *DM* diabetes mellitus, *GDF-15* growth differentiation factor 15, *IL-6* interleukin-6, SBP systolic blood pressure

^†^Multivariate analysis includes all those significant variables presented in the univariate analyses

## Discussion

The calcification process may have differing impacts on CVD outcomes due to specific pathways related to the progression of disease or calcification per se. Therefore, understanding sex differences may have implications for potential therapeutic targets and interventions. We report that in males oxidative stress (measured as AGEs in the skin) seems to be predominantly related to CAC when compared with females. However, in females, both oxidative stress and inflammation are associated with CAC. In addition, AVC seems to be driven by chronological age and inflammation in males.

AVC and CAC are both associated with increased risk for cardiovascular events [17]. Examination of the link between AVC and CAC in the Multi-Ethnic Study of Atherosclerosis (MESA) study [17] revealed that AVC increased as CAC severity increases, and that AVC is independently correlated with increased coronary atherosclerosis as assessed by CAC. Aortic sclerosis and atherosclerosis share risk factors such as hypertension, diabetes mellitus, hypercholesterolaemia, and an elevated inflammatory profile [17]. Thus, AVC may be indicative of atherosclerotic burden. CAC has been reported to correlate with aortic root calcification based on shared arterial tissue, which could indicate diffuse arterial disease, whilst the absence of a link between AVC and CAC may reflect a distinct disease [18]. Conversely, a study by Dai et al. [19], observed that there is an overlap of AVC and CAC in the KF population and AVC was associated with increased all-cause mortality regardless of the presence of CAC, conventional risk factors or inflammation. The same group also noted that the amount of AVC and CAC in various vascular calcification groups varied significantly, indicating that despite comparable risk profiles the diverse vascular beds may have distinct underlying mechanism of calcification [19]. There is still much to understand regarding the shared or differing pathophysiology of AVC and CAC.

Coronary artery calcification (CAC) is a proxy of vascular lesions that predicts atherosclerotic CVD in both CKD and non-CKD populations [20, 21]. The pooled prevalence of CAC among CKD patients has been reported to be as high as 60%, whilst in haemodialysis and renal transplant patients a pooled prevalence of 65% and 51% have been reported, respectively [22]. In addition, CAC was positively associated with an increased risk of all-cause and cardiovascular mortality [22]. Meanwhile, aortic valve calcification (AVC) has a detrimental effect on aortic valve function and gives rise to stenosis [23] that contributes to left ventricular hypertrophy and increased morbidity and mortality in CKD [10]. A recent review indicated inconsistencies in the prevalence and risk factors for vascular calcification in CKD that might be sex-dependent [24]. One study reported that males not requiring dialysis treatment have predominantly higher CAC scores than females, while other studies report a higher prevalence of aortic and carotid artery calcification in females [24]. Thus, the impact of sex influence on AVC in CKD is underrepresented.

Males presented with more prevalent CVD and greater AVC, compared to females, although the CAC score was similar in both sexes. Dyslipidaemia, with lower HDL, lipoprotein(a), and apolipoprotein A1, might explain the higher prevalence of CVD in males since it contributes to atherosclerosis [25] and higher CV event risk [26]. Additionally, the higher prescription of beta-blockers in males may also reflect a higher cardiovascular risk. Previous reports in the community-based non-CKD population showed that the choice of antihypertensive medication is sex-dependent [27]. Additionally, pharmacokinetics may play an essential role as females has lower oral beta-blocker (metoprolol) clearance and higher bioavailability [27]^.^ Thus, clinicians may be reluctant to prescribe these medications to females.

Cardiovascular calcification is an umbrella term that includes accelerated vascular ageing typical to CKD [5, 28, 29]. In this process inflammation and oxidative stress are the common triggers for vascular dysfunction and remodeling [29–31]. In our study, we find that CAC-related biomarkers had a sex-specific signature. Females with CAC were prone to be more often inflamed, with higher IL-6 and TNF levels alongside increased GDF-15 and YKL-40. These findings concur with our previous report [30] and Wang et al. [31] suggesting that chronic inflammation among CKD patients is linked to CAC. Conversely, males seemed to be subjected to more pronounced oxidative stress, measured by AGE. Whilst accumulation of AGEs are a hallmark of normal ageing, the overproduction of AGEs may lead to vascular dysfunction and structural changes via the activation of nuclear factors kappa B (NFκB) signalling [32]. This can result in coronary artery disease, even in the absence of diabetes [33, 34]. Animal studies reported higher levels of AGEs in male rats than in female rats [35]. AGE accumulation in humans may also be associated with non-traditional variables for CKD and CVD progression [36] such as cholesterol, lipoprotein(a), albuminuria, Apo-A1, and protein energy wasting as observed in females of the current study. Tanaka et al. [36] claims that racial disparities in AGE accumulation may also occur, perhaps owing to darker skin's increased absorption grade of excitation and emission light. Therefore, further investigations are required to determine if ethnic disparities or disparities in reference values between ethnicities exist among our patients, in addition to sex differences.

Despite the higher prevalence of CVD in males in the present study, the CAC score was not significantly different from that of females. RRT might obliviate the impact of sex-related factors on the vascular calcification process. The lack of a sex difference in CAC scores in our study population suggest that vascular calcification progresses occur regardless of RRT modality [37]. Owing to the small sample size, data from Jansz et al. [38] on the influence of different RRT on CAC development remain ambiguous. Niu et al. [39] also reported no significant differences in CAC development between haemodialysis and peritoneal dialysis patients. Although no sex-disaggregated analysis was undertaken their findings confirm that age and diabetes were substantially linked with CAC. However, we could see that in females RRT was associated with higher CAC score. This could also be related to the observations that haemodialysis and peritoneal dialysis contribute to sex hormone imbalance [40] by causing premature menopause in females and a decline in testosterone in males [41]. Further examination of the impact of various RRT on vascular calcification in different sexes is warranted.

It is known that the CAC score predicts all-cause mortality in CKD. CAC score was also found to be dependent on age, but not sex, in studies of CKD stages 3 to 5 [42] and advanced CKD [43]. Herein, we observe a relationship between CAC score and mortality in both males and females. After multiple adjustments this relationship of CAC score and mortality was found to be independent of age in females, in addition to hypoalbuminemia being identified as mortality predictors. However, in males after multiple adjustments, CAC score was found not to be associated with mortality and GDF-15 was identified as a potential mortality predictor, and observation we have previously reported [14]. The association of vascular calcification and KF patient mortality may be associated with a cumulative interaction of hazards such as vascular stiffness, left ventricular hypotrophy, myocardial fibrosis, and/or conductive anomalies [38]. More studies are required in larger cohorts to assess the associations more accurately between CAC and mortality in males and females.

The higher life expectancy of females than males, may be partially attributable to a lower frequency of CVD [44]. In contrast, females and males with CKD have similar life expectancies. After the initiation of RRT the sexes die at the same rate and the “cancelled survival advantage” in females is not regained even after renal transplantation [44]. This phenomenon may explain why despite male participants having more CVD than females, there was no observed difference in CAC score between the sexes. Moreover, the CAC score was associated with mortality in females in our study. It might be important to note that mortality can result from the complex interplay of other factors, such as protein energy wasting, the type and duration of RRT therapy, variation in types of surgical vascular access, as well as response and compliance to therapy.

Computed tomography is the standard technique in assessing AVC, while echocardiography (ECG) evaluates valve function. Several studies addressing AVC in CKD have used ECG for estimating calcification load and aortic valve stenosis [22, 45]. Neither a haemodialysis cohort [45] nor CKD stages 1 to 4 cohort [22] reported sex differences in AVC. In contrast, studies performed in the general population shows that severe aortic valve stenosis is more prevalent in males than females and that males are exposed to a faster progression of valvular calcification [46]. Additionally, less calcification but more fibrosis in valve biopsies is seen in female vs male valves [47, 48]. As females with AVC were underrepresented (only 14 cases), we only conducted analysis of males in whom we observed that inflammatory markers were associated with AVC. Our findings are in accordance with those reported in subjects without overt CVD [49].

The main strength of our study is a sex-disaggregated approach to the cardiovascular calcification profile in KF patients. Additionally, that we used the gold standard technique–computed tomography—to evaluate CAC and AVC. Some limitations should also be considered. At first, the low number of females with positive AVC precludes sex-stratified statistics. Moreover, we did not collect data on sex hormone status. Future investigations should include sex hormone levels or menopausal status as well as information on racial or ethnic representation. In addition, available oxidative stress markers were limited to only AGE and homocysteine in the present cohort, expansion with a wider panel of oxidative stress markers would be preferential for future investigations to properly assess the associations between oxidative stress and calcification. These may provide additional insights into the disparities or heterogeneity of findings on uremic vascular calcification.

## Perspectives and significance

In summary, we report a sex-specific signature of CAC and AVC-related biomarkers. Herein, CAC was found to be associated with oxidative stress in male KF patients, and oxidative stress and inflammation in female KF patients. In addition, in male KF patients AVC presented with an inflammatory phenotype. Our study highlights a sex-specific signature of CAC-related biomarkers that may have a potential effect on vascular complications associated with KF in males and females. Moreover, CAC was associated with mortality in females, although larger studies are warranted. Future directions include the assessment of RRT outcome concerning the sex origin of the donated kidney that may affect amelioration of vascular calcification and mortality. In addition, the impact of biological vs chronological age on the vascular calcification process needs attention. Finally, CVD phenotyping, particularly in males, should be accompanied by more frequent testing to screen for early vascular ageing and calcification.

## Data Availability

The datasets generated and/or analysed during the current study are available from the corresponding author upon reasonable request.
